# Words and Melody Are Intertwined in Perception of Sung Words: EEG and Behavioral Evidence

**DOI:** 10.1371/journal.pone.0009889

**Published:** 2010-03-31

**Authors:** Reyna L. Gordon, Daniele Schön, Cyrille Magne, Corine Astésano, Mireille Besson

**Affiliations:** 1 Center for Complex Systems and Brain Sciences, Florida Atlantic University, Boca Raton, Florida, United States of America; 2 Institut de Neurosciences Cognitives de la Méditerranée, CNRS and Université de la Méditerranée, Marseille, France; 3 Department of Psychology, Middle Tennessee State University, Murfreesboro, Tennessee, United States of America; 4 U.R.I. Octogone-Lordat 4156, Université de Toulouse II, Toulouse, France; University of Barcelona, Spain

## Abstract

Language and music, two of the most unique human cognitive abilities, are combined in song, rendering it an ecological model for comparing speech and music cognition. The present study was designed to determine whether words and melodies in song are processed interactively or independently, and to examine the influence of attention on the processing of words and melodies in song. Event-Related brain Potentials (ERPs) and behavioral data were recorded while non-musicians listened to pairs of sung words (prime and target) presented in four experimental conditions: same word, same melody; same word, different melody; different word, same melody; different word, different melody. Participants were asked to attend to either the words or the melody, and to perform a same/different task. In both attentional tasks, different word targets elicited an N400 component, as predicted based on previous results. Most interestingly, different melodies (sung with the same word) elicited an N400 component followed by a late positive component. Finally, ERP and behavioral data converged in showing interactions between the linguistic and melodic dimensions of sung words. The finding that the N400 effect, a well-established marker of semantic processing, was modulated by musical melody in song suggests that variations in musical features affect word processing in sung language. Implications of the interactions between words and melody are discussed in light of evidence for shared neural processing resources between the phonological/semantic aspects of language and the melodic/harmonic aspects of music.

## Introduction

Strong arguments have been made for both the opposing frameworks of modularity versus shared resources underlying language and music cognition (see reviews [1]–[5]). On the one hand, double dissociations of linguistic and musical processes, documented in neuropsychological case studies, often point to domain-specific and separate neural substrates for language and music [3], [6]–[9]. On the other hand, results of brain imaging and behavioral studies have often demonstrated shared or similar resources underlying, for instance, syntactic and harmonic processing [10]–[14], auditory working memory for both linguistic and musical stimuli [15], and semantic or semiotic priming [16]–[21].

These conflicting results may stem from the use of different methods, but also from other methodological problems. The main disadvantage to comparing language and music processing by testing perception of speech and musical excerpts is that the acoustic properties, context, and secondary associations (e.g., musical style or linguistic pragmatics) between even the most carefully controlled stimuli may vary greatly between the two domains. One ecological alternative is to study the perception of song [22]. In this case, linguistic and musical information are contained in one auditory signal that is also a universal form of human vocal expression. Furthermore, a better understanding of the neural basis of song is surely germane to the ongoing debate on the evolutionary origins of language and music, especially in view of propositions that the protolanguage used by early humans was characterized by singing [23], [24] and that vocal learning was a key feature governing the evolution of musical and linguistic rhythm [25]. While most studies of music cognition have used non-vocal music stimuli, everyday music-making and listening usually involve singing. Moreover, from a developmental perspective, singing is also quite relevant for parent-infant bonding, as indicated by studies showing that babies prefer infant-directed singing to infant-directed speech [26], [27].

Early studies of song cognition used dichotic listening paradigms to reveal lateralization patterns of left-ear (right hemisphere) advantage for melody recognition and right ear (left hemisphere) advantage for phoneme recognition in song [28] and in the recall of musical and linguistic content of sung digits [29]. Despite the lateralization tendencies, melody and lyrics appear to be tightly integrated in recognition [30] and priming experiments [31]. Indeed, the melody of a song may facilitate learning and recall of the words [32], [33], though this advantage appears to be diminished when the rate of presentation is controlled for, such that spoken lyrics are presented at the same rate as sung ones [34]. Furthermore, the segmentation of a pseudo-language into relevant units is facilitated for sung compared to spoken pseudowords [35], and infants learn words more easily when sung on melodies rather than when spoken [36].

The extent to which semantics and emotions are conveyed by song lyrics remains a controversial issue. One study showed that when participants were asked to listen to songs from a variety of popular music genres, they performed only at chance level when attempting to interpret the singer's intended message of each song [37]. Thus, while explicit literary interpretations of song lyrics do not appear consistent in this study, other work has suggested that sung lyrics have a greater influence over listeners' mood than the same melody played on an instrument [38]. However, this effect was amplified when the lyrics were sung with piano accompaniment, showing that the musical dimension retains importance. It has also been reported that lyrics intensify emotional responses to sad and angry music, yet mitigate the response to happy and calm music [39].

A key feature of several recent studies is the use of attentional focus to examine the interaction or independence of words and melodies in song, either by directing listeners' attention to language and music simultaneously [40]–[42], or to language only [41], [43]–[47], or to music only [41], [43]. Some of these studies have demonstrated interactive effects between the linguistic and musical dimensions of song, thereby suggesting that common cognitive processes and neural resources are engaged to process language and music. Bigand et al. [44] showed that a subtle variation in harmonic processing interfered with phoneme monitoring in the perception of choral music sung with pseudowords. In a follow-up study, the authors used a lexical decision task on sung sentence material to demonstrate that harmonic processing also interfered with semantic priming [46]. These observed interactions between semantics and harmony, measured through the implicit processing of the musical dimension, suggest that language and music in song are perceptually interwoven. Interestingly, data recently obtained by Kolinsky et al. [43] using a Garner paradigm [48] provides evidence that, while consonants remain separable from melody, vowels and melody are strongly integrated in song perception. This interaction may stem from integration of vowel and musical pitch in initial stages of sensory processing [49]. Sung sentences were also used by Fedorenko et al. [47] to demonstrate that the processing of syntactically complex sentences in language is modulated by structural manipulations in music, thereby indicating that structural aspects of language and music seem to be integrated in song perception.

By contrast, other studies of song perception and memory have shown evidence for independent processing of the linguistic and musical dimensions of song. Besson et al. [40] used the Event-Related brain Potential (ERP) method to study the relationship between words and melodies in the perception of opera excerpts sung without instrumental accompaniment. When musicians were asked to passively listen to the opera excerpts and pay equal attention to lyrics and tunes, results showed distinct ERP components for semantic (N400) and harmonic (P300) violations. Furthermore, the observed effects were well accounted for by an additive model of semantic and harmonic processing (i.e., results in the double violation condition were not significantly different from the sum of the simple semantic and melodic violations). Additional behavioral evidence for the independence of semantics and harmony in song was provided by a second experiment utilizing the same stimuli [41] and a dual task paradigm. When musician and non-musician listeners had to detect semantic and/or harmonic violations in song, results showed that regardless of musical expertise, there was no decrease in performance when listeners simultaneously attended language and music, compared to attending only one dimension at a time. These results contrast with those recently obtained by van Besouw et al. [42], showing a detriment to performance in recalling pitch contour and recalling words when listeners had to simultaneously pay attention to the words and pitch in song, as well as a similar detriment when they were asked to pay attention to the words and pitch contour of speech. Singing was also used innovatively in a series of experiments by Levy et al. [50], [51] that highlighted the influence of task demands and attentional focus on the perception of human voices in a non-linguistic context; the oddball paradigm generated a task-dependent positive ERP component (P320) in response to sung tones compared to instrumental tones.

The present study was developed to further investigate the interaction or independence of the linguistic and musical dimensions by examining the electrophysiological and behavioral correlates of words and melody in the perception of songs by individuals without formal musical training (and who are thus most representative of the general population). The choice to test non-musician participants was motivated by compelling evidence reviewed by Bigand & Poulin-Charronnat [52], in support of the idea that day-to-day normal exposure to music teaches non-musicians to implicitly process the structural aspects of music according to similar principles (although less explicitly) as individuals who have received extensive musical training. Results obtained with behavioral measures on non-musician participants demonstrate that pseudowords and intervals are processed interactively in song perception, regardless of whether listeners attend to the linguistic or to the musical dimensions [43]. Our goal was to determine whether the interactions between lyrics and tunes would also be observed when the linguistic and musical complexity of the sung stimuli was increased by using real words sung on short melodies.

**Figure 1:**
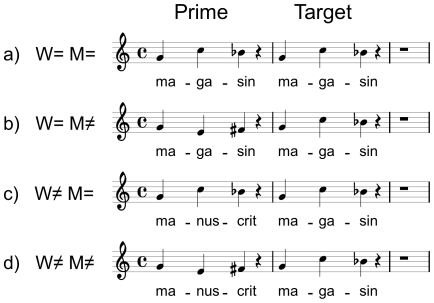
Stimuli examples. Examples of stimuli in the four experimental conditions: same word, same melody (a); same word, different melody (b); different word, same melody (c); different word, different melody (d).

The specific aim of the present experiment was two-fold: to determine the nature of the relationship (independent or interactive) between the linguistic and musical dimensions of sung words, and to specify how attention influences the dynamics of that relationship. To achieve these goals, we presented listeners with prime-target pairs of tri-syllabic words sung on 3-note melodies and recorded behavioral and electrophysiological data while they performed a same/different task. Compared to the prime, the melody and words of the sung target was manipulated orthogonally to create four experimental conditions: Same Word/Same Melody (W = M = ); Same Word/Different Melody (W = M≠); Different Word/Same Melody (W≠M = ); Different Word/Different Melody (W≠M≠; see Figure 1 for examples).

On the basis of previous findings that the N400 component is elicited by semantically unexpected or unrelated words in pairs of words [53], [54], read and spoken sentences [55]–[57], and sung sentences [40], and from results showing decreased N400 amplitude with repetition [58], we predicted that different targets, semantically unrelated to the prime (W≠), would elicit larger N400 components, slower Reaction Times (RTs) and higher error rates than same, repeated targets (W = ) [59], [60].

Besson et al. [40] also showed that an opera excerpt ending on an incongruous pitch evoked a positive component, P300/P600, typically associated with surprising events such as melodic incongruities [61]–[64]. Thus, we predicted that different melodies (M≠) would also elicit larger P300/P600 components, and slower RTs and higher error rates [65], compared to same melody (M = ).

Finally, if the perception of words and melodies in songs call upon independent processes, the Word effect (different – same word) should be similar, in behavioral measures and N400 amplitude, for same and different melodies. Likewise, the Melody effect (different – same melody) should be similar, in behavioral measures and P300/P600 amplitude, for same and different words. If the perception of words and melodies in sung words rely instead on interactive processes, the Word effect should be different for same and different melodies (interference effects) and vice-versa for the Melody effect. In addition, the use of an orthogonal design allows us to test the additive model following which the ERP in the double variations condition (W≠M≠) should be equivalent to the sum of the ERPs in the simple variations conditions (W≠M =  plus W = M≠).

In order to determine how attention to one dimension or another modulates the processing of words and melody in song, we asked participants to perform a same/different task on the same set of stimuli and to focus their attention either on the linguistic dimension (Linguistic Task: are target words same or different as prime words?) or on the musical dimension (Musical Task: are target melodies same or different as prime melodies?). The same-different task has been used extensively in the literature to investigate the relationship between two dimensions of a stimulus in various modalities (e.g., melody recognition [66]; letter recognition [67]; meaningful environmental sounds [68]), and is particularly effective when participants are asked to attend to only one dimension at a time (see Thomas [69] for a review and in-depth analysis of the same-different task).

## Methods

### A. Participants

Twenty-one volunteers (15 females; mean age = 25 years old; age range 18–32) were paid 16 euros to participate in this experiment that lasted for about 90 minutes including preparation time. Informed consent was obtained from all participants, and the data was analyzed anonymously. Verbal consent was used because at the time of data collection, the local ethics committee did not require written consent for experiments using behavioral or ERP methods in healthy adult individuals. This study was approved by the CNRS - Mediterranean Institute for Cognitive Neuroscience and was conducted in accordance with local norms and guidelines for the protection of human subjects. All participants had normal hearing, no known neurological problems, and were native French-speaking, right-handed non-musicians (all had less than two years of formal music lessons).

### B. Stimuli

We created a set of 480 different pairs of stimuli (primes and targets). First, a list of 120 pairs of French tri-syllabic nouns was established. In each pair, the prime and target words were different and semantically unrelated. The phonological and phonetic characteristics of the words were controlled and we limited the use of certain phonemes with intrinsically longer durations (e.g. fricatives [70]), as well as consonant clusters, so that syllabic duration would be as consistent as possible between words. To increase task difficulty and to homogenize the linguistic and musical dimensions, the first syllable and the first note of the prime and target within a pair were always the same.

Next, 120 pairs of different 3-note isochronous melodies were created while controlling the harmonic content and using all 12 keys. All intervals up to the major sixth were used except the tritone. The melodic contour was also balanced across the stimuli. One quarter of the melodic pairs (30 melodies) consisted of a prime with rising contour (defined as two successive ascending intervals) paired with a target with falling contour (defined as two successive descending intervals) and vice versa for another ¼ of the pairs. The other half of the pairs consisted of “complex” contours: ¼ of the pairs had a prime made up of an ascending interval plus a descending interval, followed by a target with a descending plus an ascending interval, and vice-versa for the last ¼ of the pairs. These different types of contours were evenly distributed among the experimental conditions. No melody was used more than three times, and any melody appearing more than once was always transposed into a different key and paired with a different prime melody. The melodies were written in a vocal range that was comfortable for the singer.

Finally, the pairs of melodies were randomly assigned to the pairs of words. Once the 120 different pairs had been created, they were distributed evenly over the four experimental conditions: W = M = ; W = M≠; W≠M =  and W≠M≠ with 30 trials per condition (see Figure 1 and supporting materials Audio S1, Audio S2, Audio S3, Audio S4 for stimulus examples, and the Appendix S1 for a list of stimuli used). In order to control for specific stimulus effects, 4 lists were constructed so that each target appeared in all 4 conditions across the 4 lists (Latin square design).

The 120 targets and 480 primes were sung *a capella* by a baritone. Recording sessions took place in an anechoic room. In order to prevent listeners from making judgments based solely on lower-level acoustic cues, two different utterances of the sung words were selected to constitute the pairs in the W = M =  conditions (in natural speech/song no two pronunciations of a segment by the same speaker are ever identical, but listeners normalize over perceived segments [71]). Although the singer sung at a tempo of 240 beats per minute to control syllable duration, natural syllabic lengthening always occurred on the last syllable/note, giving rise to an average duration of all stimuli of 913 ms (SD = 54 ms). All words were normalized in intensity to 66 dB (SD across items = 1 dB).

### C. Procedure

Participants listened, through headphones, to 120 pairs of sung words from the four experimental conditions presented in pseudorandom order. The same pairs were presented twice in two attentional tasks: Linguistic and Musical. In the Linguistic task, participants were instructed to pay attention only to the language in order to decide, by pressing one of two response keys as quickly and accurately as possible, if the two words were the same or different. In the Musical Task, participants were instructed to pay attention only to the music in order to decide, as quickly and accurately as possible, if the two melodies were the same or different.

Each session began with a block of practice trials. Each trial consisted of a prime sung word followed by a target sung word, with an SOA of 1800 ms. Participants were asked to avoid blinking until a series of X's appeared on the computer screen at the end of each trial. Response keys, order of tasks, and stimuli lists were counterbalanced across participants. The software Presentation (Neurobehavioral Systems, Albany, CA) was used to present stimuli and record behavioral responses (RTs and % errors).

### D. Data acquisition

EEG was recorded continuously from 32 “active” (pre-amplified) Ag-AgCl scalp electrodes (Biosemi, Amsterdam) and located according to the International 10/20 system. The data were re-referenced offline to the algebraic average of the left and right mastoids. In order to detect eye movements and blinks, the horizontal electrooculogram (EOG) was recorded from electrodes placed 1 cm to the left and right of the external canthi, and the vertical EOG was recorded from an electrode beneath the right eye. The EEG and EOG signals were digitized at 512 Hz and were filtered with a bandpass of 0.1–40 Hz (post-analysis data were filtered with a lowpass of 10 Hz for visualization purposes only). Data were later segmented in single trials of 2200 ms starting 200 ms (baseline) before target onset. Trials containing ocular or movement artifacts or amplifier saturation (determined by visual inspection) were excluded from the averaged ERP waveforms (i.e., on average 12% of the trials, thereby leaving approximately 26 out of a possible 30 trials in each condition per participant). Individual data analysis and grand averages were computed using the Brain Vision Analyzer software (Brain Products, Munich).

### E. Data Analyses

Behavioral data (RTs and arcsin-transformed Error Rates) were analyzed using a three-way ANOVA with within-subject factors: Attentional Task (Linguistic vs. Musical), Word (same vs. different), and Melody (same vs. different). A four-way ANOVA with factors Task Order, Attentional Task, Word, and Melody was computed to determine if results were influenced by the order in which participants performed the two tasks: Linguistic task first or Musical Task first. Although a main effect of Order was found, showing that the second task (whether Linguistic or Musical) was performed better than the first task (thereby reflecting increased familiarity with the experimental procedure), no significant interactions of Order with other factors were found, so this factor was not considered further.

Mean amplitude ERPs to the target words were measured in several latency bands (50–150, 150–300, 300–500, 600–800, 800–1000 ms) determined both from visual inspection and from results of consecutive analyses of 50-ms latency windows from 0 to 2000 ms. Eight regions of interest were defined by first separating the electrodes into two groups: midlines (8) and laterals (24), and then defining subsets of electrodes for analysis. The midlines were divided into two regions of interest: fronto-central: (Fz, FC1, FC2, Cz) and parieto-occipital (CP1, CP2, Pz, Oz). The lateral electrodes were separated into 6 regions of interest: left frontal (FP1, AF3, F3, F7), left temporal (FC5, T7, CP5, C3), left parietal (P3, P7, PO3, O1), right frontal (FP2, AF4, F4, F8), right temporal (FC6, T8, CP6, C4) and right parietal (P4, P8, PO4, O2). For the midline electrodes, an ANOVA with factors Attentional Task (Linguistic vs. Musical), Word (same vs. different), Melody (same vs. different) and Region (fronto-central vs. parieto-occipital) was computed on the mean amplitudes of the ERPs in each latency band. A similar ANOVA was computed for the lateral electrodes, with Attentional Task, Word, Melody, Hemisphere (left vs. right) and Region (frontal vs. temporal vs. parietal) as factors. Results of the ANOVAs are reported only when significant at p<0.05. All p values for ERP results were adjusted with the Greenhouse-Geisser epsilon correction for nonsphericity when necessary. For both behavioral and ERP results, when interactions between two or more factors were significant, pairwise post-hoc comparisons between relevant condition pairs were computed and thresholded by Bonferroni correction. When post-hoc analysis revealed that none of the simple effects constituting an interaction reached the threshold for Bonferroni significance, the interaction was not considered further.

## Results

### Behavioral data

Mean Reaction times and Error rates are reported in Table 1.

**Table 1:** Behavioral data.

	Linguistic Task				Musical Task			
Condition	W = M =	W = M≠	W≠ M =	W≠ M≠	W = M =	W = M≠	W≠ M =	W≠ M≠
RTs	718 (151)	756 (162)	786 (131)	783 (153)	919 (168)	1003 (153)	1129 (221)	1109 (255)
% Err	0.8 (1.5)	0.6 (1.3)	1.0 (2.1)	1.1 (1.9)	0.8 (1.8)	3.7 (5.3)	3.5 (4.5)	8.9 (9.1)

Mean Reaction Times (RTs) and errors rates (in %) for each of the 4 experimental conditions (W = M = : same word, same melody; W = M≠: same word, different melody; W≠M = : different word, same melody; W≠M≠: different word, different melody), in the Linguistic and Musical tasks. SD is indicated in parentheses.

The ANOVA on RTs showed that participants were slower in the Musical Task (1040 ms) than in the Linguistic Task (761 ms; main effect of Task [*F*(1,20) = 72.26, p<0.001]). Moreover, RTs were slower for W≠ (952 ms) than W =  (849 ms; main effect of Word [*F*(1,20) = 88.46, p<0.001]). Finally, the Task x Word interaction was significant [*F*(1,20) = 22.76, p<0.001]: in the Musical Task participants were slower for W≠ (1119 ms) than for W =  (961 ms; simple effect of Word: posthoc p<0.001) but this difference was not significant in the Linguistic Task. The Task x Melody interaction was not significant but the Word x Melody interaction was significant [*F*(1,20) = 18.44, p<0.001]: RTs were slower for M≠ (879 ms) than for M =  (818 ms) only when words were same (W = ; posthoc p<0.001). By contrast, RTs were slower for W≠ than for W =  regardless of whether melodies were same (M = ) or different (M≠, both posthoc p's<0.001).

The ANOVA on Error rates showed that participants made more errors in the Musical Task (4.21%) than in the Linguistic Task (0.87%) [main effect of Task: *F*(1,20) = 20.95, p<0.001]. Moreover, both the Task x Word and the Task x Melody interactions were significant [F(1,20) = 9.53, p = 0.006 and F(1,20) = 9.21, p = 0.006, respectively]. In the Musical Task participants made more errors for W≠ (6.19%) than for W =  (2.22%; simple effect of Word: posthoc p<0.001) and for M≠ (6.27%) than for M =  (2.14%; simple effect of Melody: posthoc p<0.001), but these differences were not significant in the Linguistic Task. The Word x Melody interaction was not significant.

### ERP data

**Figure 2:**
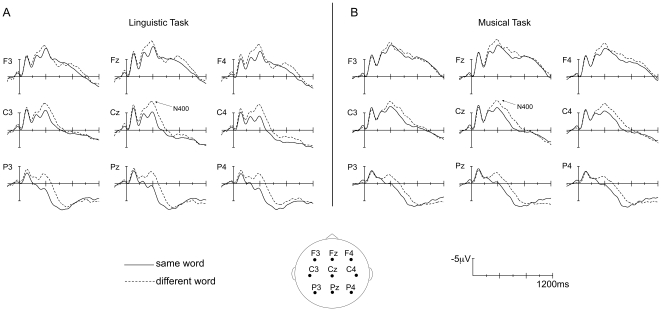
Word effect. Grand average ERPs timelocked to the onset of targets with the same word as the prime (solid line) or a different word than the prime (dashed line), in the Linguistic Task (A) and Musical Task (B). Selected traces from 9 electrodes are presented. In this figure, amplitude (in microvolts) is plotted on the ordinate (negative up) and the time (in milliseconds) is on the abscissa.

**Figure 3:**
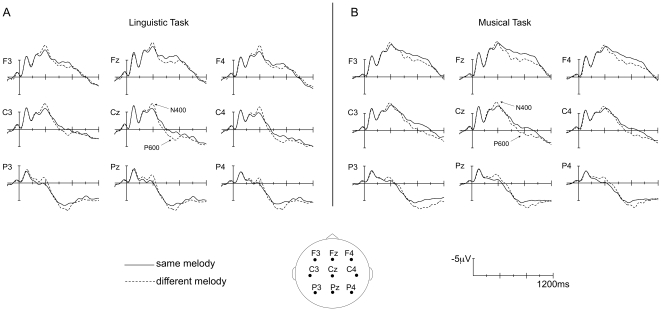
Melody effect. Grand average ERPs timelocked to the onset of targets with the same melody as the prime (solid line) or a different melody than the prime (dashed line), in the Linguistic Task (A) and Musical Task (B). Selected traces from 9 electrodes are presented. In this figure, amplitude (in microvolts) is plotted on the ordinate (negative up) and the time (in milliseconds) is on the abscissa.

Results of the ANOVAs on ERP data in the different latency ranges are presented in Table 2. When the main effects or relevant interactions were significant, results of pairwise posthoc comparisons are reported in the text (except for posthoc results of the Word by Melody interaction, which are reported in Table 3). The Word effect and the Melody effect in each task are illustrated on Figures 2 and 3, respectively.

**Table 2:** ANOVA results on mean amplitudes of ERPs.

			Latency (ms)									
			50–150		150–300		300–500		600–800		800–1000	
	Factors	df	F	p	F	p	F	p	F	p	F	p
**Midlines**	W	1,20			5.89	0.025	50.10	<0.001	5.51	0.029		
	M	1,20					6.78	0.017	10.99	0.004	7.58	0.012
	T×W	1,20	4.9†	0.039†			5.53	0.029				
	W×R	1,20					21.31	<0.001				
	M×R	1,20							6.52	0.019		
	W×M	1,20					7.14	0.015				
	T×W×R	1,20					4.65	0.044				
**Laterals**	W	1,20			4.40	0.049	28.08	<0.001				
	M	1,20					7.06	0.015	6.08	0.023		
	T×W	1,20	8.85†	0.008†	4.90†	0.039†	15.60	<0.001			4.52†	0.046†
	W×R	2,40					26.01	<0.001				
	W×M	1,20					7.19	0.014				
	W×H	1,20									5.79	0.026
	W×H×R	2,40	6.33	0.007	4.76	0.018	3.88	0.036				
	T×W×R	2,40					3.98	0.046				
	T×M×R	2,40							3.58	0.048		

Results of ANOVAs computed on midline and lateral electrodes for main effects, 2-way and 3-way interactions. Only significant effects (p<0.05) are shown. Abbreviations: df, degrees of freedom; T, Attentional Task; W, Word; M, Melody; H, Hemisphere; R, Region.

† Pairwise comparisons of interest did not meet the criteria for Bonferroni significance and thus the interaction is not discussed further in the text.

**Table 3:** Posthoc comparisons for Word x Melody interaction.

Pairwise Comparison	Midlines	Laterals
W = M = vs. W = M≠	0.006*	0.004*
W = M = vs. W≠M =	<0.001*	<0.001*
W = M = vs. W≠M≠	<0.001*	<0.001*
W = M≠ vs. W≠M =	0.004*	0.032
W = M≠ vs. W≠M≠	0.02	0.092
W≠M = vs. W≠M≠	0.493	0.596

Results of pairwise posthoc comparisons for the Word x Melody interaction, in the 300–500 ms latency band. Pairs that meet the criteria for significance with the Bonferroni threshold (p = 0.0083) are indicated with *.

*Between 50 and 150 ms*, different words (W≠) elicited a larger N100 component than Same words (W = ) over the right frontal region (Word x Hemisphere x Region interaction; p<0.001). This effect was larger in the Linguistic Task than in the Musical Task at lateral electrodes (p = 0.021; see Figure 2), but this result did not reach significance after Bonferroni correction.

*Between 150 and 300 ms*, W≠ elicited a smaller P200 component than W =  (main effect of Word at both midline and lateral electrodes). This effect was more prominent over bilateral frontal and left parietal regions (Word x Hemisphere x Region; all p<0.001). Again, this effect was larger in the Linguistic than in the Musical Task at lateral electrodes (p = 0.011; see Figure 2) but this result was only marginally significant with the Bonferroni correction.

*Between 300 and 500 ms*, W≠ elicited a larger N400 component than W =  at both midline and lateral electrodes (main effect of Word), with larger differences over parieto-occipital than fronto-central midline electrodes (Word x Region interaction: both p<0.001), and over parietal and temporal lateral regions (Word x Region, both p<0.001), with a slight right hemisphere predominance (Word x Hemisphere x Region, both p<0.001). The N400 effect (W≠ minus W = ) was larger at lateral electrodes in the Linguistic (p<0.001) than in the Musical Task (p = 0.004; Task x Word) and at midlines (both p<0.001), with a centro-parietal scalp distribution in the Linguistic Task and a parietal distribution in the Musical Task (Task x Word x Region at midline and lateral electrodes, all p<0.001).

**Figure 4:**
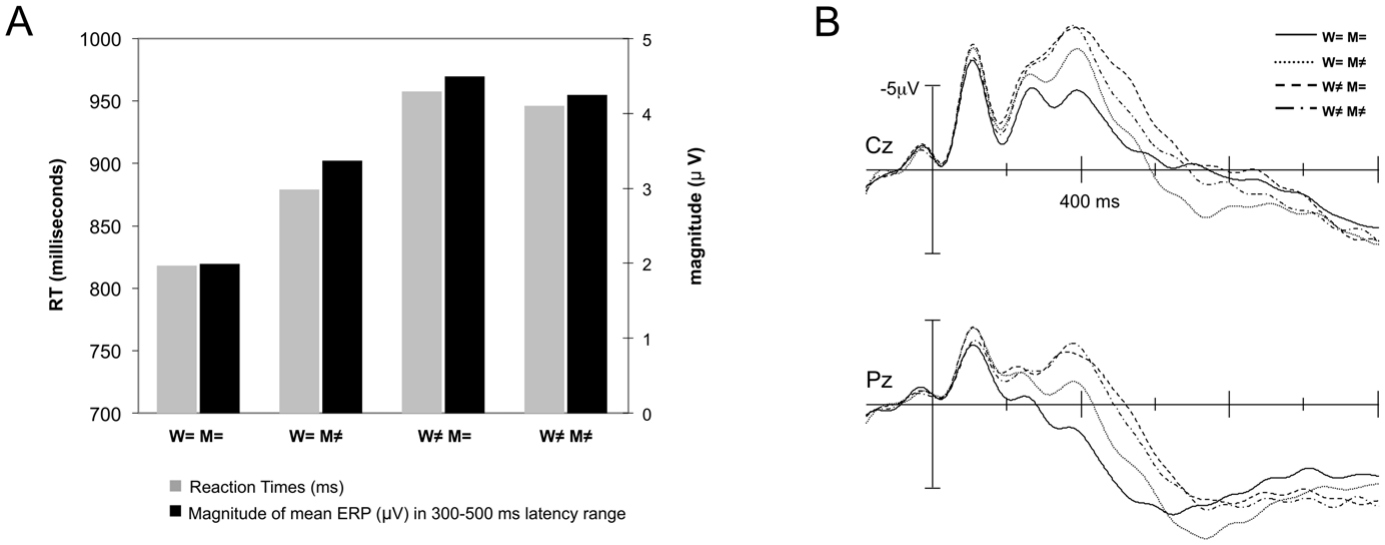
Word by Melody interaction. (A) For each of the 4 experimental conditions (averaged across both tasks because there was no Task x Word x Melody interaction): the reaction time in milliseconds (gray bars, left Y-axis) and the magnitude (µV) of the mean amplitude of the ERPs in the 300–500 ms latency range, averaged across all electrodes (black bars, right Y-axis). (B) ERPs associated with the 4 experimental conditions (averaged across both tasks because there was no Task x Word x Melody interaction) for electrodes Cz (top) and Pz (bottom). Solid line: same word, same melody; dotted line: same word, different melody; dashed line: different word, same melody; dashed-dotted line: different word, different melody.

M≠ elicited larger N400-like components than M =  (main effect of Melody at midline and lateral electrodes; see Figure 3). Moreover, the Word x Melody interaction was significant at midline and at lateral electrodes: the Melody effect (M≠ vs. M = ) was only significant when Word was same (W = ) but not when Word was different (W≠; see Table 3 for all posthoc p-values for the Word x Melody interaction). Likewise, the Word effect was only significant when Melody was same (M = ) but not when Melody was different (M≠; see Figure 4, which shows the four orthogonal conditions averaged over both tasks). Furthermore, negative components in the W = M≠, W≠M = , and W≠M≠ conditions were larger than in W = M =  condition. At the midline electrodes, negative components were also larger in the W≠M =  than in the W = M≠ conditions.

**Figure 5:**
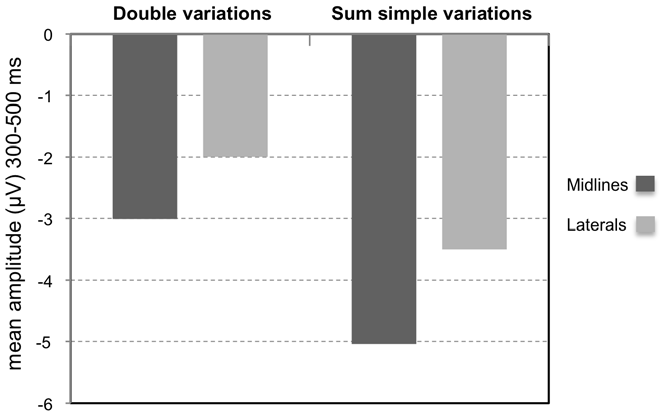
Additive model test. Mean amplitude (in µV) of ERP difference waves in the 300–500 ms latency band, for double variations observed (W≠M≠ minus W = M = ) and the modeled sum of simple variations (W≠M =  minus W = M = ) + (W = M≠ minus W = M = ), at midline electrodes (dark gray bars) and lateral electrodes (light gray bars).

To further test the Word by Melody interaction, difference waves were computed (on mean amplitudes) for each of the following comparisons: *d1 = * W≠M =  minus W = M =  (effect of Word when Melody is same); *d2 = * W = M≠ minus W = M =  (effect of Melody when Word is same); *d3* = W≠M≠ minus W = M =  (effect of different Word and different Melody). If words and melodies are processed independently, then *d1*+*d2* should be equal to *d3*. ANOVAs with factor Data (double variation condition [*d3*] vs. additive model [*d1+d2*]) together with the other factors of interest (for midlines: Attentional Task and Region and for laterals: Attentional Task, Hemisphere, and Region) were carried out. Results showed that the sum of the ERP effects of the simple variations (d1 + d2) was significantly larger than the ERP effects in the double variations condition [d3; midline electrodes, *F*(1,20) = 7.14, p = 0.015; lateral electrodes, *F*(1,20) = 7.19, p = 0.014]; see Figure 5.

*Between 600 and 800 ms*, W≠ still elicited more negative ERPs than W =  (main effect of Word at midline electrodes) but M≠ elicited larger late positive components than M =  (main effect of Melody at midline and lateral electrodes, see Figure 3). At the midline electrodes, this effect was larger over the fronto-central region than the parieto-occipital region (both p<0.001; Melody x Region); furthermore, at lateral electrodes, the effect was larger over temporal and parietal regions (both p<0.001) in the Linguistic Task but was larger over frontal regions (p<0.001) in the Musical Task (Task x Melody x Region).

*Between 800 and 1000 ms*, W≠ still elicited larger negativities than W =  over the right hemisphere (p = 0.002; Word x Hemisphere). This effect was larger in the Linguistic than in the Musical Task (p = 0.017) but this difference did not reach significance with the Bonferroni correction. Finally, M≠ still elicited larger positive components than M =  (main effect of Melody at midline electrodes).

### Scalp distribution of the N1, P2, and N400 components (Word effects)

ERPs in the N1, P2, and N400 latency bands were more negative for different word than for same word. These effects may consequently reflect an early onset of the N400 effect, or three distinct components. Since different scalp distributions were found in each of the three latency bands tested separately, it was therefore of interest to directly compare the Word effect (W≠ minus W = ) across latency bands. To this end, we conducted additional ANOVAs on the difference waves, with factors: Latency Band (50–150 ms vs. 150–300 ms vs. 300–500 ms), Hemisphere (left vs. right), and Region (frontal vs. temporal vs. parietal). Results showed a significant Latency band x Region interaction [*F*(4,80) = 43.15, p<0.001]. While there were no significant differences in scalp distribution between the effect of Word in the 50–150 ms (N1) and in the 150–300 ms (P2) latency bands, the topography of the N400 (300–500 ms) was different from both the N1 and the P2. Pairwise posthoc comparisons showed that the N400 had a more parietal distribution compared to the N1 (p<0.001) and the P2 (p<0.001). The Latency x Hemisphere x Region interaction was not significant.

In order to prevent the topographical shape of the ERPs from being potentially confounded by the amplitude of ERP effects, the same statistical analysis was then repeated on data that had undergone vector scaling (c.f. [72], but see also [73] for a discussion of the limitations of this method). The Latency x Region interaction was again significant [*F*(4,80) = 21.22, p<0.001], and pairwise posthoc tests showed the same pattern of results as in the unscaled data. This analysis therefore confirmed that the frontal distribution of the early negativities (N1/P2 complex) is significantly different from the parietal distribution of the N400.

## Discussion

### Processing the words

As predicted on the basis of several results in both the behavioral (e.g., [59]) and neurolinguistic literatures (e.g., [40], [54], [55], [57]), sung word targets that were different from sung word primes (W≠) were associated with lower levels of performance (more errors and slower RTs) and with larger N400 components than same words (W = ). Thus, as noted in [40], similar processes seem to be involved in accessing the meaning of spoken and sung words. One could argue that access to word meaning was not necessary to perform the Linguistic Task and that participants could have based their decision on phonological cues. However, this is unlikely as previous work on spoken words has demonstrated that word meaning is processed automatically in phonological tasks [74], [75], prosodic tasks [76]–[78], during passive listening in the waking state [74], [75], and even during sleep [79].

Moreover, the finding that an N400 word effect also developed in the Musical Task, with similar onset latency and duration (until around 800 ms post-target onset), and a similar scalp distribution in the 300–500 ms latency range as in the Linguistic Task (centro-parietal for language and parietal for music; see Figure 2), also provides evidence in favor of the automatic processing of sung word meaning regardless of the direction of attention. The smaller size of the N400 effect in the Musical than in the Linguistic Task was most likely due to fewer attentional resources being available for processing words in the Musical Task (attention focused on the melody) than in the Linguistic Task (attention focused on words), as has been argued previously [75], [76], [78].

Early Word effects were also found with larger N100 components in the 50–150 ms latency band and smaller P200 components in the 150–300 ms latency band over frontal regions to different (W≠) than same words (W = ; see Figure 2). Even though both same and different words started with the same first syllable, which lasted for 250 ms on average, subtle articulation differences (in particular, in vowel quality and pitch of the sung syllable) were most likely present in the first syllable of different target words (e.g., the “me” in “messager” does not sound identical to the “me” in “mélodie”). Moreover, even though the post-hoc comparison for the Task by Word interaction was not significant after Bonferroni correction between 50–150 ms and between 150–300 ms (probably because task differences were too small), it is clear from Figure 2 that the N100 and P200 effects were primarily present when participants attended to the words. Attending to the linguistic dimension may have amplified participants' sensitivity to small differences in co-articulation, which in turn influenced the early perception of sung words, just as subtle phonetic differences modulate the N100 in speech perception [80]. This interpretation is supported by the vowel harmony phenomenon described by Nguyen & Fagyal [81], in which the pronunciation of the vowel of the first syllable assimilates to the anticipated vowel of the second syllable, which was indeed different in the W≠ conditions. We also considered the idea that the early N100 and P200 effects were the leading edge of the N400 component, in light of previous reports demonstrating the early onset of the auditory N400 effect [82], possibly reflecting the fact that lexico-semantic processing starts before the spoken word can be fully identified [83]. However, this interpretation seems unlikely in view of the results of the scalp distribution analysis that demonstrated a significant difference between the frontally-distributed early negativities and parietally-distributed N400.

### Processing the melody

Different melodies (M≠) compared to same melodies (M = ) elicited larger negative components between 300 and 500 ms, followed by larger late positive components in the 600–1000 ms latency band.

The P600 component was expected based on previous reports showing that unexpected melodic/harmonic variations (e.g., [61]–[64], [84]) elicit effects belonging to the P300 family of components. These effects are generally interpreted as reflecting the processing of surprising and task-relevant stimuli [85]–[87] and are indicative of the allocation of attention and memory resources (see Polich [88] for a recent review and discussion of functionally divergent P3 subcomponents). The longer onset latency of the positive effect in the present experiment than in previous studies is probably due to the fact that the first note of the melody was the same in both the M≠ and M =  conditions, with the second note being sung at around 250 ms post-onset of the target. Interestingly, the task did influence the scalp distribution of the late positivity, which was frontal when the melodies were explicitly processed (Musical Task) and parietal when the melodies were implicitly processed (Linguistic Task). The frontal scalp distribution of the positive component in the Musical Task is consistent with the scalp distribution of the P3a component reported for chord sequences ending with dissonant harmonies [84] and harmonically acceptable chords with deviant timbre [89]. The parietal scalp distribution of the positive component in the Linguistic Task is consistent with previous results when participants were asked to pay attention to both lyrics and tunes [40].

Finally, it is interesting to note that late positivities, i.e., the late positive potential (LPP), have also been observed during the evaluation of affective stimuli [90], [91], such as tones sung with a sad voice presented simultaneously with sad pictures [92]. In the present study, the musical dimension of the sung words, although minimal, may have called upon emotional processes, reflected by the late positivities. Further work on the emotional response to singing may clarify these issues.

One of the most interesting findings of the present study is that, prior to the late positive components, M≠ also elicited widely distributed, larger negative components than M =  in the 300–500 ms latency band in both the Linguistic and Musical tasks (see Figure 3). This negativity bears the scalp distribution and peak latency typically seen for the N400 component. Indeed, N400's have been recently associated with musical incongruities related to memory and emotional meaning, such as in familiar melodies containing an unexpected but harmonically congruous note [66], or when a mismatch ensues between musical chords and emotion words (e.g., a dissonant chord target primed by the visually presented word “love”) [18]. However, the N400 Melody effect in the present study was slightly smaller in amplitude than the N400 Word effect at the midline electrodes. The difference between these effects may be due to an overlap with the subsequent late positive component generated in the M≠ but not in the W≠ condition, but could also result from greater intrinsic salience of the linguistic dimension in songs [30], [31].

Thus, in both attentional tasks, words sung on different melodies (M≠) were associated with larger N400 components than words sung on same melodies (M = ). Since the intonational contour of lyrics in song is provided by the musical melody, it has been suggested that the variations in prosodic-like effects for sung lyrics could explain why words in song are better recognized with their original melodies than with a different melody [93]. In fact, several recent studies show that words spoken with prosodically incongruous patterns are associated with increased amplitudes of the N400 component followed by late positivities [78], [94], [95]. Thus, words sung on different melodies may hinder lexical access in a similar manner as unexpected prosodic patterns in spoken language. If familiarity is established through repeated listening to a song, which may reinforce prosodic representations of the words that are created by the melody, then the present findings may be better understood in light of results obtained by Thompson & Russo [45]. They showed that participants perceived the meaning of song lyrics as enhanced when familiarity with the songs was increased (see section 6.4 in [5] for an interesting discussion of those results). We could thus speculate that our participants' *lexico-semantic expectations for sung words* were violated not only when the target word was different from the prime (W≠M =  condition) but also when the target melody was different from the prime (W = M≠). This interpretation accounts for the N400 effects associated with differences on each dimension as they stand in contrast to the tight perceptual combination of repeated words and melodies (W = M = ). Further work is needed to differentiate how variations in the musical dimension of songs affect lexical access [96], general semantic memory [97], and conceptual relatedness [20], [21], [98]. For instance, future studies using pairs of sung words that are semantically related to each other, or sung word targets primed by other meaningful stimuli (e.g. pictures, environmental sounds, or meaningful musical excerpts), could elucidate the dynamics of the N400 component in song.

Overall, results showed that N400 components are generated when the target does not match the prime in pairs of sung words on either dimension (linguistic or musical). It must be emphasized here that these results were found regardless of the direction of attention, thereby reflecting the automatic processing of the linguistic and musical dimensions when words are sung. This pattern of results may also reflect the inability of participants to selectively focus their attention on the words or on the melodies, precisely because the two dimensions cannot be separated. We explore this possibility next.

### Interactive processing

Both behavioral and ERP data in the N400 latency band clearly revealed interactive processing of the linguistic and musical dimensions in song, which occur simultaneously in sung words. This interaction was found independently of the direction of attention (i.e., in both the Linguistic and Musical tasks and furthermore in the absence of a Task by Word by Melody interaction). Moreover, results of an ANOVA on the difference waves did demonstrate that the theoretical sum of the ERPs for simple linguistic and musical variations was significantly larger than the actual ERP in the double variation condition (see also Figure 5). Therefore, an additive model did not account for the data reported here. Furthermore, the pattern of interaction is strikingly symmetric between the two dimensions. The N400 word effect (different vs. same words) only occurs when melodies are the same; likewise, the N400 melody effect (different vs. same melodies) and the effect on RTs (slower for M≠ than M = ) only occur when words are same but not when words are different, as illustrated in Figure 4. These findings coincide with previous studies of sung and spoken language that have documented an influence of the musical dimension on linguistic processing, even when attention is directed to the linguistic aspect [43], [44], [46], [47], [99]. Thus, the main conclusion that can be drawn from these results is that words and melody are closely interwoven in early stages of cognitive processing. This outcome is compatible with a recent report by Lidji et al. [49] of ERP evidence for interactive processing between vowel and pitch in song perception. The spatio-temporal brain dynamics of this integrated response could be responsible for interactive effects between word and melody in song, observed in a growing number of behavioral studies on perception [43], [44], [46], [47], learning [35], [36], and memory [30]–[33].

Some important differences between our protocol using sung word pairs and previous studies using opera excerpts [40], [41] can provide an explanation for why we did not find the same tendency toward independence of neural and behavioral correlates associated with the perception of words and melodies. First, the type of same-different task employed in the present study on stimulus pairs, but not in [40] and [41], has been previously used by Miranda & Ullman [66] to show that notes that are tonally congruous (in-key) but incorrect in familiar melodies elicit both the N400 and P600 components, even when participants' attention was directed away from pitch. Furthermore, the violation paradigm used by Besson et al. [40] and Bonnel et al. [41], in which the last note of the sung phrase of the opera excerpt was not only unexpected in the context but also out-of-key, may have made wrong notes more salient for the listener than the more subtle different melody targets used in the present experiment. Indeed, even when the target melody was different than the prime, it contained tonal intervals in a reduced harmonic context. In fact, subtle stimulus variations have been used in several studies reporting interaction of linguistic and musical processing, such as the interference of harmony on phonological and semantic processing [44], [46] or the interaction of semantics and harmony [17].

Nevertheless, it should be noted that the present results also provide some evidence for separate effects associated with the linguistic and musical dimensions. First, RTs were slower for different than same words regardless of whether melodies were same or different (but, as mentioned above, RTs were slower for different than for same melodies only when words were same). This slightly asymmetric pattern of interferences may be related to the fact that our non-musician participants were less accustomed to making explicit judgments about melodic information than linguistic information, as demonstrated by slower RTs in the Musical Task than in the Linguistic Task. These results correspond to those obtained in the first of a series of experiments on non-musicians by Kolinsky et al. [43] showing slower reaction times in the melodic than phonological task, in addition to an enhanced interference effect between phonology and intervals in the melodic task.

Second, while early differences were found in the 50–150 and 150–300 ms latency bands were found between same and different words (independently of the melodies), no such early differences were observed between same and different melodies. As discussed above, these early differences mostly likely reflect an effect of co-articulation caused by phonetic differences already present in the first syllable of different words rather than an early onset of the N400 word effect.

Finally, differences in the late positivity were found between same and different melodies but not between same and different words. As mentioned above, results of several experiments have shown increased P3 components to unexpected variations in melody or harmony [40], [61]–[64], [84], [89], typically interpreted as reflecting the allocation of attention and memory resources to task-relevant stimuli [85]–[88]. The late positivity in the present study may also be related to the LPP, which is associated with the processing of affective stimuli [90]–[92]. Based on these accounts, the absence of a difference in late positive components for words may reflect the fact that they were easier to process than melodies (thereby requiring fewer attentional and memory resources) or that they did not elicit an emotional response. This last interpretation could be tested in further experiments by using affective sung words as targets.

To summarize, the present results show that N400 components were elicited not only by different words but also by different melodies, although the effect of melody began later and was followed by a late positive component. Moreover, the effects of melody and word were interactive between 300 and 500 ms, thereby showing that lyrics and tunes are intertwined in sung word cognition. A companion study conducted in our lab with the fMRI method, using the same stimuli and attentional tasks, also yielded robust interactions between words and melody in songs in a network of brain regions typically involved in language and music perception [100]. These results are consistent with a growing number of studies establishing that language and music share neural resources through interactive phonological/semantic and melodic/harmonic processing (cf. [5]).

The present findings, along with other recent work on song perception and performance, are beginning to respond to the question of why song is, and has been since prehistoric times [23], [24], so prevalent in the music perception and performance activities occurring in most humans' daily lives. Intrinsic shared mechanisms between words and melody may be involved in a number of song-related behaviors that have shaped human nature, although we do not yet know if the linguistic-musical interactions are the cause or effect of these tendencies. For example, it appears that infants' preference for singing over speech [26] cannot be merely attributed to the presence of the musical dimension [27] and may reflect a specific proclivity for singing-based mother-infant interactions. In early humans, adding melody to speech may have fostered parent-infant bonding and thus given an evolutionary advantage to individuals possessing more highly developed musical traits [101]. Singing to children fosters language acquisition, perhaps because exaggerated prosody aids segmentation [102] and the added musical information provides redundant cues for learning [35], [36]. Melody in song may also serve as a mnemonic for storage of words in long-term memory (e.g., [33]). Research along these lines may also begin to shed light on the mechanisms responsible for the benefits of Melodic Intonation Therapy and other singing-based music therapy techniques in the speech rehabilitation process [103].

## Supporting Information

Audio S1 Example of stimulus pair in condition same word/same melody (W =  M = ).
(0.22 MB WAV)

Audio S2 Example of stimulus pair in condition same word/different melody (W =  M≠).
(0.22 MB WAV)

Audio S3 Example of stimulus pair in condition different word/same melody (W≠ M = )
(0.22 MB WAV)

Audio S4 Example of stimulus pair in condition different word/different melody (W≠ M≠).
(0.22 MB WAV)

Appendix S1 Pairs of sung words in each of the four experimental conditions, in one list of the Latin Square design (the first author can be contacted to obtain the other three lists), with each trisyllabic French word and the 3-note melody on which it was sung (one note per syllable). The melodies are represented in standard MIDI codes, where: C4 = 60, C#4 = 61, D4 = 62, D#4 = 63, E4 = 64, F4 = 65; F#4 = 66; G4 = 67; G#4 = 68; A4 = 69; A#4 = 70; B4 = 71, C5 = 72, and so on.
(0.24 MB DOC)
