# Supplementary material for: Words and Melody Are Intertwined in Perception of Sung Words: EEG and Behavioral Evidence
Source: PLoS One. 2010 Mar 31;5(3):e9889. doi: 10.1371/journal.pone.0009889 (PMC2847603; doi:10.1371/journal.pone.0009889)
Supplement: Appendix S1 — Pairs of sung words in each of the four experimental conditions, in one list of the Latin Square design (the first author can be contacted to obtain the other three lists), with each trisyllabic French word and the 3-note melody on which it was sung (one note per syllable). The melodies are represented in standard MIDI codes, where: C4 = 60, C#4 = 61, D4 = 62, D#4 = 63, E4 = 64, F4 = 65; F#4 = 66; G4 = 67; G#4 = 68; A4 = 69; A#4 = 70; B4 = 71, C5 = 72, and so on. (0.24 MB DOC) [file pone.0009889.s005.doc]

**Same Word, Same Melody (W=M=)**

| **prime word** | **sung on prime melody** | | |  | **target word** | **sung on target melody** | | |
| --- | --- | --- | --- | --- | --- | --- | --- | --- |
| agonie | 66 | 70 | 71 |  | agonie | 66 | 70 | 71 |
| arrivée | 64 | 67 | 72 |  | arrivée | 64 | 67 | 72 |
| avocat | 62 | 67 | 69 |  | avocat | 62 | 67 | 69 |
| boulanger | 64 | 66 | 71 |  | boulanger | 64 | 66 | 71 |
| cinéma | 68 | 70 | 72 |  | cinéma | 68 | 70 | 72 |
| colonel | 64 | 65 | 69 |  | colonel | 64 | 65 | 69 |
| confetti | 61 | 60 | 58 |  | confetti | 61 | 60 | 58 |
| député | 60 | 58 | 57 |  | député | 60 | 58 | 57 |
| énoncé | 63 | 60 | 59 |  | énoncé | 63 | 60 | 59 |
| enseignant | 66 | 63 | 59 |  | enseignant | 66 | 63 | 59 |
| étudiant | 63 | 61 | 58 |  | étudiant | 63 | 61 | 58 |
| harmonie | 66 | 69 | 70 |  | harmonie | 66 | 69 | 70 |
| horizon | 67 | 60 | 64 |  | horizon | 67 | 60 | 64 |
| industrie | 67 | 60 | 58 |  | industrie | 67 | 60 | 58 |
| institut | 65 | 60 | 58 |  | institut | 65 | 60 | 58 |
| liberté | 65 | 57 | 58 |  | liberté | 65 | 57 | 58 |
| monarchie | 61 | 59 | 60 |  | monarchie | 61 | 59 | 60 |
| mutation | 63 | 59 | 61 |  | mutation | 63 | 59 | 61 |
| numéro | 63 | 58 | 61 |  | numéro | 63 | 58 | 61 |
| officier | 64 | 60 | 59 |  | officier | 64 | 60 | 59 |
| possession | 68 | 61 | 66 |  | possession | 68 | 61 | 66 |
| président | 69 | 61 | 66 |  | président | 69 | 61 | 66 |
| procédé | 66 | 57 | 59 |  | procédé | 66 | 57 | 59 |
| qualité | 60 | 68 | 63 |  | qualité | 60 | 68 | 63 |
| sentiment | 63 | 70 | 65 |  | sentiment | 63 | 70 | 65 |
| symphonie | 64 | 68 | 66 |  | symphonie | 64 | 68 | 66 |
| téquila | 64 | 72 | 71 |  | téquila | 64 | 72 | 71 |
| tourbillon | 65 | 70 | 67 |  | tourbillon | 65 | 70 | 67 |
| unité | 66 | 70 | 67 |  | unité | 66 | 70 | 67 |
| vagabond | 67 | 72 | 71 |  | vagabond | 67 | 72 | 71 |

**Same Word, Different Melody (W=M≠)**

| adhésion | 66 | 69 | 71 |  | adhésion | 66 | 61 | 57 |
| --- | --- | --- | --- | --- | --- | --- | --- | --- |
| appétit | 68 | 59 | 63 |  | appétit | 68 | 70 | 69 |
| canapé | 63 | 61 | 60 |  | canapé | 63 | 68 | 72 |
| casino | 62 | 59 | 57 |  | casino | 62 | 64 | 71 |
| citoyen | 67 | 64 | 59 |  | citoyen | 67 | 69 | 70 |
| combattant | 68 | 72 | 69 |  | combattant | 68 | 61 | 63 |
| comité | 69 | 72 | 71 |  | comité | 69 | 60 | 62 |
| diffusion | 61 | 57 | 58 |  | diffusion | 61 | 70 | 66 |
| division | 64 | 57 | 59 |  | division | 64 | 68 | 67 |
| émotion | 65 | 72 | 67 |  | émotion | 65 | 61 | 63 |
| estomac | 63 | 58 | 59 |  | estomac | 63 | 70 | 67 |
| faculté | 64 | 65 | 72 |  | faculté | 64 | 61 | 57 |
| habitant | 61 | 58 | 59 |  | habitant | 61 | 69 | 66 |
| héritier | 67 | 59 | 60 |  | héritier | 67 | 72 | 68 |
| inconnu | 64 | 57 | 60 |  | inconnu | 64 | 72 | 65 |
| maladie | 68 | 66 | 59 |  | maladie | 68 | 71 | 72 |
| mélodie | 65 | 70 | 68 |  | mélodie | 65 | 57 | 62 |
| mobilier | 61 | 65 | 70 |  | mobilier | 61 | 59 | 57 |
| océan | 67 | 71 | 72 |  | océan | 67 | 65 | 58 |
| pavillon | 65 | 70 | 72 |  | pavillon | 65 | 64 | 57 |
| pétrolier | 66 | 71 | 68 |  | pétrolier | 66 | 59 | 64 |
| pilotis | 62 | 61 | 59 |  | pilotis | 62 | 65 | 70 |
| position | 62 | 70 | 65 |  | position | 62 | 57 | 60 |
| précision | 65 | 63 | 61 |  | précision | 65 | 69 | 72 |
| randonnée | 66 | 58 | 65 |  | randonnée | 66 | 70 | 68 |
| résultat | 63 | 67 | 72 |  | résultat | 63 | 62 | 60 |
| résumé | 66 | 67 | 69 |  | résumé | 66 | 62 | 57 |
| saladier | 67 | 62 | 58 |  | saladier | 67 | 69 | 71 |
| vidéo | 62 | 67 | 70 |  | vidéo | 62 | 58 | 57 |
| volupté | 64 | 71 | 67 |  | volupté | 64 | 60 | 61 |

**Different Word, Same Melody (W≠M=)**

| adoption | 62 | 57 | 58 |  | accusé | 62 | 57 | 58 |
| --- | --- | --- | --- | --- | --- | --- | --- | --- |
| alphabet | 69 | 70 | 72 |  | alchimie | 69 | 70 | 72 |
| embarras | 64 | 72 | 67 |  | ambition | 64 | 72 | 67 |
| balançoire | 66 | 68 | 69 |  | bâtiment | 66 | 68 | 69 |
| campagnard | 63 | 60 | 58 |  | candidat | 63 | 60 | 58 |
| chaudronnier | 64 | 63 | 59 |  | chocolat | 64 | 63 | 59 |
| compagnie | 64 | 68 | 71 |  | continent | 64 | 68 | 71 |
| dépression | 67 | 72 | 69 |  | délégué | 67 | 72 | 69 |
| édition | 66 | 58 | 61 |  | écolier | 66 | 58 | 61 |
| échéance | 69 | 71 | 70 |  | écurie | 69 | 71 | 70 |
| extension | 63 | 71 | 68 |  | exposé | 63 | 71 | 68 |
| figurant | 65 | 58 | 62 |  | financier | 65 | 58 | 62 |
| gratitude | 62 | 60 | 61 |  | gravité | 62 | 60 | 61 |
| impulsion | 65 | 69 | 68 |  | intérêt | 65 | 69 | 68 |
| imprudence | 60 | 63 | 70 |  | invention | 60 | 63 | 70 |
| latitude | 66 | 68 | 71 |  | lavabo | 66 | 68 | 71 |
| monopole | 60 | 57 | 59 |  | modestie | 60 | 57 | 59 |
| autobus | 68 | 72 | 70 |  | oreiller | 68 | 72 | 70 |
| pancréas | 67 | 65 | 60 |  | pantalon | 67 | 65 | 60 |
| papillon | 64 | 71 | 68 |  | paradis | 64 | 71 | 68 |
| pénurie | 68 | 65 | 58 |  | pédalo | 68 | 65 | 58 |
| politesse | 63 | 66 | 70 |  | poésie | 63 | 66 | 70 |
| restaurant | 63 | 65 | 72 |  | rescapé | 63 | 65 | 72 |
| rotation | 69 | 64 | 60 |  | romancier | 69 | 64 | 60 |
| satelitte | 63 | 61 | 59 |  | salarié | 63 | 61 | 59 |
| société | 65 | 57 | 64 |  | solution | 65 | 57 | 64 |
| soulagement | 65 | 58 | 60 |  | soumission | 65 | 58 | 60 |
| tétanos | 68 | 64 | 59 |  | théorie | 68 | 64 | 59 |
| vacancier | 61 | 68 | 70 |  | vanité | 61 | 68 | 70 |
| vitamine | 60 | 57 | 58 |  | villageois | 60 | 57 | 58 |

**Different Word, Different Melody (W≠M≠)**

|  |  |  |  |  |  |  |  |  |
| --- | --- | --- | --- | --- | --- | --- | --- | --- |
| aliment | 65 | 60 | 57 |  | amitié | 65 | 69 | 70 |
| archétype | 61 | 57 | 59 |  | artisan | 61 | 68 | 63 |
| acouchement | 62 | 67 | 71 |  | atelier | 62 | 61 | 57 |
| canari | 66 | 59 | 57 |  | cabinet | 66 | 68 | 70 |
| collection | 65 | 63 | 58 |  | comédie | 65 | 68 | 72 |
| descendant | 64 | 59 | 62 |  | démission | 64 | 72 | 69 |
| domicile | 68 | 60 | 67 |  | document | 68 | 71 | 69 |
| énergie | 67 | 69 | 72 |  | éléphant | 67 | 62 | 59 |
| écrivain | 65 | 62 | 58 |  | équation | 65 | 67 | 70 |
| habileté | 64 | 69 | 72 |  | haricots | 64 | 62 | 57 |
| inertie | 61 | 63 | 70 |  | illusion | 61 | 58 | 57 |
| inflation | 63 | 72 | 68 |  | incendie | 63 | 58 | 60 |
| hypothèse | 62 | 57 | 59 |  | ironie | 62 | 70 | 69 |
| jalousie | 62 | 60 | 57 |  | japonais | 62 | 66 | 71 |
| manuscrit | 67 | 64 | 66 |  | magasin | 67 | 72 | 70 |
| nucléaire | 62 | 65 | 72 |  | nudité | 62 | 60 | 59 |
| omoplate | 62 | 57 | 61 |  | opinion | 62 | 71 | 69 |
| partition | 68 | 72 | 71 |  | pardessus | 68 | 60 | 63 |
| paradoxe | 65 | 72 | 69 |  | passager | 65 | 61 | 62 |
| protégé | 67 | 64 | 57 |  | promotion | 67 | 68 | 72 |
| cantatrice | 64 | 71 | 69 |  | quantité | 64 | 59 | 63 |
| recrutement | 67 | 63 | 58 |  | religion | 67 | 70 | 71 |
| relation | 62 | 60 | 58 |  | renouveau | 62 | 69 | 71 |
| séduction | 65 | 67 | 72 |  | scénario | 65 | 62 | 57 |
| syndicat | 66 | 57 | 61 |  | sympathie | 66 | 71 | 70 |
| tarentule | 61 | 57 | 60 |  | tabouret | 61 | 69 | 62 |
| tradition | 64 | 68 | 65 |  | tragédie | 64 | 59 | 61 |
| vallonnement | 65 | 70 | 66 |  | variation | 65 | 61 | 64 |
| vigilance | 67 | 70 | 69 |  | vibration | 67 | 58 | 62 |
| vocation | 65 | 67 | 69 |  | volonté | 65 | 61 | 58 |
